# Worth one’s salt

**DOI:** 10.1007/s12471-018-1191-4

**Published:** 2018-10-18

**Authors:** A. E. Schaafsma, E. A. van der Have, H. Lameijer

**Affiliations:** Department of Emergency Medicine, Medical Centre Leeuwarden, Leeuwarden, The Netherlands

A 37-year-old male patient was brought to the emergency department by emergency medical services. His past medical history included a suicide attempt by medication overdose. Physical examination showed shallow breathing with normal oxygenation, a heart rate of 117 bpm, blood pressure of 116/78 mm Hg, capillary refill time of 3 s and a Glasgow Coma Scale score of 1-1-1 (E-M-V); the pupils were wide, equal and reactive to light. During the emergency department visit the patient suffered three epileptic seizures, which were treated with midazolam intravenously. After emergency endotracheal intubation by the emergency physician, an electrocardiogram was performed (Fig. 1). Which abnormalities should be a further guide to the diagnosis in this patient? How should this patient be treated?

**Fig. 1:**
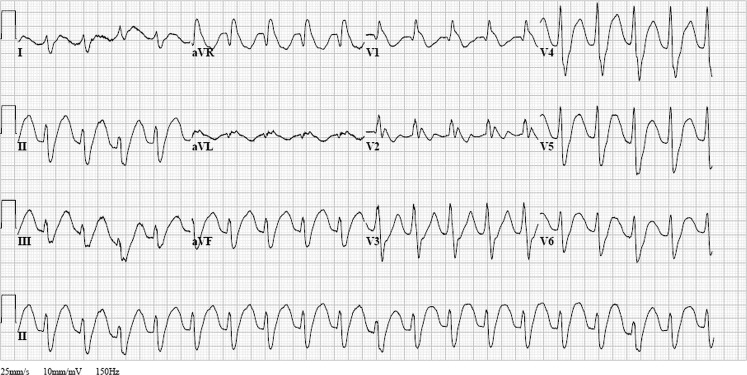
Electrocardiogram performed at the emergency department

## Answer

You will find the answer elsewhere in this issue.

